# Clinical evaluation and resting state fMRI analysis of virtual reality based training in Parkinson’s disease through a randomized controlled trial

**DOI:** 10.1038/s41598-022-12061-3

**Published:** 2022-05-16

**Authors:** Farzin Hajebrahimi, Halil Aziz Velioglu, Zubeyir Bayraktaroglu, Nesrin Helvaci Yilmaz, Lutfu Hanoglu

**Affiliations:** 1 Functional Imaging and Cognitive-Affective Neuroscience Lab (fINCAN), Research Institute for Health Sciences and Technologies (SABITA), Istanbul Medipol University, Istanbul, Turkey; 2 Department of Physical Therapy and Rehabilitation, Institute of Health Sciences, Istanbul Medipol University, Istanbul, Turkey; 3 Department of Physical Therapy and Rehabilitation, School of Health Sciences, Istanbul Medipol University, Istanbul, Turkey; 4 Present Address: Department of Women’s and Children’s Health, Karolinska Institute, Stockholm, Sweden; 5 Department of Physiology, International School of Medicine, Istanbul Medipol University, Istanbul, Turkey; 6 Department of Neurology, School of Medicine, Istanbul Medipol University, Istanbul, Turkey

**Keywords:** Parkinson's disease, Movement disorders

## Abstract

There are few studies investigating the short-term effects of Virtual Reality based Exergaming (EG) on motor and cognition simultaneously and pursue the brain functional activity changes after these interventions in patients with Parkinson’s Disease (PD). The purpose of this study was to investigate the synergistic therapeutic effects of Virtual Reality based EG on motor and cognitive symptoms in PD and its possible effects on neuroplasticity. Eligible patients with the diagnosis of PD were randomly assigned to one of the two study groups: (1) an experimental EG group, (2) an active control Exercise Therapy (ET) group. All patients participated in a 4-week exercise program consisting of 12 treatment sessions. Every session lasted 60 min. Participants underwent a motor evaluation, extensive neuropsychological assessment battery and rs-fMRI before and after the interventions. Thirty patients fulfilled the inclusion criteria and were randomly assigned to the EG and ET groups. After the dropouts, 23 patients completed the assessments and interventions (11 in EG, 13 in ET). Within group analysis showed significant improvements in both groups. Between group comparisons considering the interaction of group × time effect, showed superiority of EG in terms of general cognition, delayed visual recall memory and Boston Naming Test. These results were consistent in the within-group and between-group analysis. Finally, rs-fMRI analysis showed increased activity in the precuneus region in the time × group interaction in the favor of EG group. EG can be an effective alternative in terms of motor and cognitive outcomes in patients with PD. Compared to ET, EG may affect brain functional connectivity and can have beneficial effects on patients’ cognitive functions and motor symptoms. Whenever possible, using EG and ET in combination, may have the better effects on patients daily living and patients can benefit from the advantages of both interventions.

## Introduction

Despite a promising effect of pharmacological treatments on motor symptoms, no definitely known treatment is suggested regarding the non-motor symptoms in patients with Parkinson’s Disease (PD)^1^. Nonpharmacological interventions have been introduced as a complementary treatment in addressing symptoms of PD. Nonpharmacological interventions include treatments such as physiotherapy, cognitive rehabilitation, treadmill training, exercise therapy and are shown to be effective in various outcomes in PD^2^. Addressing motor and nonmotor symptoms has been shown to have beneficial effects on clinical outcomes in patients with PD^3^. While adherence to the pure motor or cognitive treatment can be a challenge in the clinical facilities, technological interventions such as Virtual Reality (VR) and Exergaming (EG), may help to increase the patients’ motivation and attending rehabilitation sessions more effectively and be a promising tool to address both motor and nonmotor symptoms of patients with PD simultaneously. This idea may ignite the hypotheses that VR and EG interventions may be more effective by addressing both motor and nonmotor symptoms and these effects can be captured in the brain activity networks. Effects of EG on clinical outcomes has been shown in the previous PD studies^4–6^. Similarly, cognitive rehabilitation designed for attention and executive dysfunctions have been shown to modify functional connectivity (FC) in the frontoparietal (executive) network and the Default Mode Network (DMN)^7,8^. However, the patients may not be so interested to take part in a mere cognitive training session^9^. Lastly, V-Time study here remains one of the largest studies, which investigated the effects of adding virtual feedback to the treadmill training and reported the promising effects on brain activation levels^10^.

The results of PET studies show that cognitive impairment in PD is characterized by a decreased metabolism in prefrontal, temporal and parietal regions and increased metabolism in cerebellum^11^. As cognitive impairment moves towards dementia, the hypometabolism can spread to the anterior cingulate cortex^12^. Interestingly, resting state-functional Magnetic Resonance Imaging (rs-fMRI) studies confirm this result and affirm that hypoactivation in the anterior cingulate cortex can also be seen in the early stages of the disease, even without emerged dementia^13^. Therefore, the changes in brain functional activities can be divided into two stages: (1) in PD patients without cognitive deficits, hyperconnectivity can be seen and accepted as an effort to use all additional brain capacity to compensate progressive cell loss in PD^14^; (2) as disease progresses and cognitive decline occurs, hypoconnectivity can be seen^15,16^. Moreover, the results of a study that used Independent Component Analysis (ICA) on both fMRI and FDG-PET data of healthy controls and PD patients with and without Mild Cognitive Impairment (MCI), revealed that glucose metabolism was significantly reduced in all DMN nodes in both patient groups when compared to controls, and FC within the DMN was accompanied by similar changes in metabolic connectivity and associated with metabolic deficits in PD patients with and without MCI^17^.

Although applications like EG are so affordable and easy to use in patients with PD, their effects on brain networks remains relatively unclear. There is a lack of studies investigating the short-term effects of VR based EG on motor and cognition simultaneously and pursue the brain functional activity changes after these interventions. rs-fMRI, which measures the brain activity in the presence of no task, has been shown to be an important method in detecting effects of neurorehabilitation^18^.

The purpose of this Randomized Controlled Trial was to investigate the synergistic therapeutic effects of VR based EG on motor and cognitive symptoms in PD and its possible effects on neuroplasticity. We hypothesized that EG training, by providing a real time feedback, increasing motivation and adherence, and engaging patients mentally in the training process, would be more effective than conventional therapy in terms of improvements in motor and cognitive symptoms, and resting state networks connectivity.

## Materials and methods

### Ethical approval

The protocol of this study was approved by the institutional Non-invasive Clinical research Ethics Committee of Istanbul Medipol University (10840098-604.01.01-E.20325). An oral and written informed consent was obtained from all the participants. All methods were performed in accordance with the principles of the Declaration of Helsinki.

### Study design

**Figure 1:**
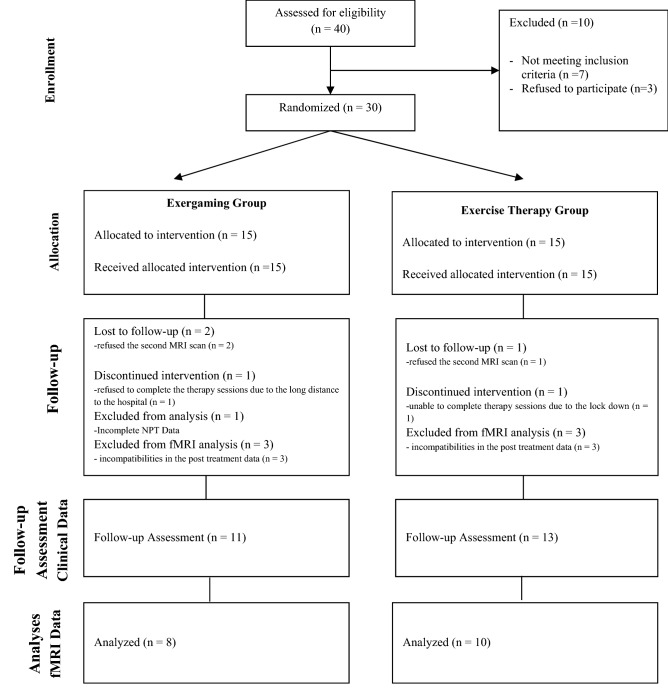
Flowchart of the study.

This study was a single-blind randomized controlled trial. Participants were randomly allocated into two groups. Participants were not aware of the interventions in other groups and were randomly assigned to one of the two study groups: (1) an experimental EG group, (2) an active control ET group. All patients participated in a 4-week exercise program consisting of 12 treatment sessions. Every session lasted 60 min. This study was adhered to the CONSORT guidelines (Fig. 1)^19^ and registered in clinicaltrials.gov (Identifier: NCT03637023, First registration Date: 17/08/2018).

### Study setting

Participants who applied to the neurology outpatient polyclinic at Istanbul Medipol University Hospital were checked for their eligibility to be included in the study. All the assessments and interventions were conducted at the neuroscience laboratory of the Hospital. MRI Acquisitions were performed at the same hospital. All assessments and interventions took place in the “ON” period of the disease. Assessment sessions were conducted 1 week before (T0) and 2 weeks after (T1) the intervention. The intervention and related assessments were conducted by an experienced physical therapist and neuropsychological tests were conducted by a neuropsychologist. Total daily dopa and equivalent dopa agonist doses received by the patients were calculated by the recommendations reported by Fenelon et al.^20^. Participants continued their medical treatment under the supervision of the neurologist and their medication was not modified during the study period.

### Participants

Patients with the diagnosis of PD were checked for their eligibility and were included in the study. Inclusion criteria were clinical diagnosis of PD within the framework of Brain Bank criteria^21^, 50 years old and older, Hoehn and Yahr stages I–III^22^ and being able to walk at least 5 min unassisted; and receiving a stable anti-parkinsonian medication for at least 1 month (or the treatment has not changed).

Exclusion criteria were having a story of unstable medical condition, history of head trauma, stroke, or exposure to toxic substances; being diagnosed with Dementia; PD patients with Freezing of Gait, implying Parkinson plus syndromes in neurological examinations; pyramidal, cerebellar examination findings, gaze paresis and autonomic dysfunction; problems in the vision or hearing; patients with device-aided treatment (i.e. DBS, infusional therapy); Presence of major depression diagnosis contraindication for the MRI scanning.

### Interventions

Patients on both groups received 4 weeks of exercises, 3 times per week. Participants in the EG group received EG intervention (12 sessions in total). EG was applied using Nintendo Wii (NW) balance board. NW Fit Plus games covering gait, static and dynamic balance were utilized during this intervention^23^. Evidence shows that using the NW is more effective than other VR game-based devices which are commonly used (like Xbox) for treating balance problems in PD^24^. Wii Fit balance board can track center of pressure while patients stand on it. A virtual environment was displayed in front of the patients, and with avatar technology, images were projected through a projector on the wall (1 × 1.6 m viewed from 3 m distance). Instant visual and audio feedback was provided^25^. By simulating the virtual character, patients were able to adjust their movements with a real time feedback (there is a negligible delay of < 20 ms between person and avatar movement). Main selection criteria for the games were applicability and safety^6^. Participants in the ET group received balance and gait training basically focusing on lower extremity movements (12 sessions in total). ET and EG applications were performed in individualized sessions and no group session was performed in neither group. The participants in the EG group performed EG with the same device for the whole intervention period under supervision of the Physical Therapist. The participants in the ET group underwent ET under supervision of the Physical Therapist in the individualized sessions. The intervention program in each session in both groups are as follows:

#### EG Group

- **Yoga games (10 min):** Focused on stretching exercises and included games such as Sun Salutation, Chair Pose and Half Moon.
- **Strengthening games (15 min):** Focused on strengthening exercises and included games such as Single Leg Extension, Torso and Waist Twist, Lung and Side Lung. Each activity repeated 10–15 times and were applied in 3 sets.
- **Balance games (35 min):** Focused on static and dynamic balance exercises. During these games, the patients were expected to adapt their centers of mass as quickly and accurately as possible. Balance games included games like Marble Balance, Ski Slalom and Balance Bubble.

Natural breathing was highlighted to prevent hypertension during exercise in all the stages.

#### ET Group

- **Stretching exercises (10 min):** Light joint flexion and extension and trunk rotation in standing position with focus on upper and lower extremities.
- **Strengthening exercises (15 min):** Focused mostly on the lower extremity muscles that are important for posture, balance, and gait. The following exercises were be given to the participants in the standing position:
  - Swing each leg forward/lateral
  - Climbing up and down the step
  - Leg lifting in different directions
  - Walking on the heel and toes
  - Squat
- **Balance Exercises (35 min):** were given as a combination of static and dynamic balance training and sensory integration training.
  - ***Static and dynamic balance exercises:***
    - Balance on the toe tips
    - Standing with feet together
    - Stand in tandem and semi-tandem position
    - Slow and fast weight transfer
    - Ball catching in different directions
    - Stepping forward and laterally in standing position
  - ***Sensory integration training:***
    - Stand on single and double feet with eyes open and closed
    - Stand on soft ground with eyes open and closed

Each exercise was repeated 10–15 times in 3 sets. Participants used ankle weights starting from 1 kg and gradually increased to 2 kg for each leg. Natural breathing was highlighted to prevent hypertension during exercise.

Successful patients in the beginner levels of the EG group were encouraged to progress into the advanced levels of the games to challenge them with more difficult motor and cognitive demands. Progression in the ET group was achieved by adding more weight to the feet, increasing the number of repetitions in each exercise, and modifying the severity of the exercises. Progression criteria was determined by perceived effort (Borg scale perceived effort < 13) and the ability of patients to perform the activity without difficulty.

### Outcomes

Participants underwent a motor evaluation, extensive neuropsychological assessment battery (NPT) and rs-fMRI before the interventions. Participants then were randomly assigned into either of the interventions. All the assessments were repeated 2 weeks after the interventions.

Motor evaluation included assessment of general disease level using the Unified Parkinson's Disease Rating Scale-motor (UPDRS-III)^26^, balance using Berg Balance Scale (BBS) and Activities-specific Balance Confidence (ABSC)^27,28^, mobility using Timed Up and Go Test (TUG)^29^, and functional capacity using Six Minute Walk Test (6MWT)^30^.

NPT included testing general cognition using Montreal Cognitive Assessment (MoCA)^31^ and five cognitive domains; (i) memory functions, using verbal memory processes test (VMPT)^32^ and visual subtest of the Wechsler Memory Scale^33^; (ii) executive functions, using Stroop Color and Word Test^34^ and Verbal Fluency (VF) Test^35^; (iii) visuospatial functions, using Benton’s Face Recognition Test (BFR) and Benton Line Judgment Orientation Test^36^; (iv) attention, using Digit-Span (DS) Forward and Backward test^37^; and (v) language skills, using Boston Naming Test (BNT)^38^. The following aspects were evaluated beside NPT: behavioral mood using Geriatric Depression Scale (GDS)^39^ and quality of life (QoL) using The Parkinson's Disease Questionnaire (PDQ-39)^40^.

rs-fMRI acquisition was performed for all the participants at the time of the motor and cognitive assessments before and after the intervention.

### MRI acquisition

Structural and functional MRI studies were performed at Istanbul Medipol University Hospital (Bagcilar, Istanbul) with a 3 T Philips Ingenia CX MR machine with 32 channels head coil using parallel imaging sequences. The participants were informed about the nature and dangers of the MR environment and trained to act accordingly. This is important especially in patients with movement disorders^41^. To minimize the head motions, spongy pads were used to fix the patients head inside the head coil before starting the scanning and the patients were asked to stay still as much as affordable. The resting state imaging scan was planned first in the imaging queue to get benefit of the high level of alertness subjects experience at the beginning of the MRI sessions. The participants were asked not to think about any specific thought and not count or perform any specific, rhythmic mental activity. After resting state sequence patients were suggested to close their eyes and rest, to minimize the discomforts, as the opening or closing eyes would not affect the anatomical scanning. Patients were scanned during their “On” period. The scanning sequences were as follows: (1) Survey, (2) Resting State, (3) Fieldmap, (4) T1W, (5) T2W. fMRI scan parameters included 300 volumes (TR 2230 ms, TE 30 ms, FA 77°) (TR/TE: 2230/30 ms), FOV 240 × 240 × 140 mm (RL × AP × FH), voxel size 3 × 3 × 4 mm, flip angle 77°, and slices 35. The Parameters for the anatomical T1 image of the sagittal section were 190 slices; (TR/TE: 8.1/3.7), FOV 256 × 256 × 190 mm (FH × AP × RL), voxel size was determined as 1 × 1 × 1 mm. Due to the different scan protocols, the second scan differed with the above-described parameters in terms of TR and Volume (TR 2000 ms, volume 341). However, all the participants had the same TR and Volume value in the pre and post scans. These values were demeaned and used as covariates of no interest in the GLM design.

### Analysis

#### fMRI data analysis

We performed a mode-free approach in analyzing fMRI data. The tools in the FMRIB FSL software package were used for pre-processing and statistical analysis steps of the anatomic and functional MRI data. Anatomical and functional images collected in DICOM format were converted to 3D and 4D single images in compressed NIFTI + format. The FSL BET tool was used to extract brain tissue from the anatomical head images after correcting the density differences due to signal inhomogeneity^42^. The head movements that occurred during fMRI recording were corrected with FSL MCFLIRT algorithm^42^. For interpersonal and group comparisons, the anatomical and functional data were spatially normalized to the MNI152 standard head model. Signal losses and voxel displacements deriving from tissue inhomogeneities were corrected using magnetic field maps acquired after the functional scan. ICA was performed on the functional data of each subject with the MELODIC tool in the FSL package for cleaning movement, physiological (heart, respiratory etc.) and other artifacts from functional data. The spatial distribution, spectral content and time series of the components were used to characterize those components compatible with the signal artifacts. The ICA components with artefacts were marked manually and regressed out from the functional data using FSL regfilt function. Group ICA analysis was performed with the MELODIC tool in FSL package on the preprocessed and cleaned data. In this analysis, independent components common at group level to resting-state activity were obtained. The common ICA components were used to calculate individual spatial component maps and time series of the subjects by FSL dual regression tool. For statistical comparisons, a GLM design matrix was created in which the difference between the pre-intervention and post-intervention status of the 2 groups was compared. Non-parametric permutation tests (5000 permutations) with FSL randomize tool were used to determine statistically significant differences between the groups. For multiple comparisons, TFCE (threshold-free cluster enhanced) technique was used for correcting for multiple comparisons^43,44^. Levodopa equivalent daily dose (LEDD) was calculated^45^. LEDD and age were included as covariates in the GLM design. All covariate values were demeaned and used as covariates of no interest. The design of the GLM used here helped to include all factors simultaneously so that the differences of two interventions could be detected regarding LEDD value and age. Therefore, the results were achieved despite the neurodegeneration effect of the PD and age factor on brain FC. Finally, in the dual regression process Findlab templates were used and activity pattern were obtained (https://findlab.stanford.edu/functional_ROIs.html)^46^.

#### Sample size

The sample size was determined using the G*power sample size calculator. Sample size was calculated using repeated measure design with an 80% power (α = 0.05, β = 0.20) and effect size of 0.35 for a sample size of 24 participants.

#### Randomization

The randomization sequence was generated once for the study using MATLAB rand.m function that reinitialized at the time of generation with current time. Sex or age equivalence between subjects in each of the groups and between the groups was not applied. Patients were not aware of the other intervention group. The neurologists and evaluators were also blind to the patient’s assignment to the groups. Only the physical therapist who conducted the interventions were aware of the patients’ groups due to the nature of the study.

### Statistical analysis

IBM SPSS (Statistical Package for Social Science) version 25.0 was used for statistical analysis. Mean, standard deviation and percentage values were presented in the descriptive statistics of the data. The normal distribution of the variables was measured with the Kolmogorov Smirnov test. The nominal data of the independent variables were evaluated with the Chi-square test, and the numerical data were evaluated with the Independent Sample *t* test. Time-dependent differences within groups and Time × Group interactions between groups were analyzed by Two-Way Repeated Measure ANOVA. The significance value was accepted as p < 0.05.

## Results

### Clinical results

Forty individuals with PD were initially screened in the pre-inclusion assessments; of these, 30 participants fulfilled the inclusion criteria and were randomly assigned to the EG (15 participants) and ET (15 participants) groups. Of 30 participants 7 did not complete the study protocol or did not attend the post treatment assessment. Therefore, 23 participants completed all the assessments and interventions (11 participants in the EG group and 13 participants in the ET group-Fig. 1). The average age of the individuals in the EG group (n = 11) was 66.36 ± 8.04. Participants were minimum 55 years old and maximum 79 years old. The average age of the individuals in the ET group (n = 13) was 65.53 ± 9.93. Participants were from a minimum of 52 to a maximum of 81 years old. 2 of the 11 individuals in the EG group were women, and 3 of the 13 individuals in the ET group were women. None of the participants reported any adverse effects. All participants in both groups were right-handed. There was no statistically significant difference in terms of age, sex, dominant hand, and education level between groups. The demographic data of the participants are shown in Table 1.

**Table 1:** Distribution of demographic data.

	EG group (n = 11)	ET group (n = 13)	t/χ^2^	p value
Age (Avg ± SD)	66.36 ± 8.04	65.53 ± 9.93	− 0.290	0.772
**Sex**				
Female (n/%)	2/18.2	3/23.1	− 0.288	0.773
Male (n/%)	9/81.8	10/76.9		
Left (n/%)	0/0	0/0		
**Education level**				
1–8 years	5/45.5	10/76.9	− 1.213	0.225
8–12 years	3/27.3	0/0		
< 12 years	3/27.3	3/23.1		

*Avg* average, *SD* standard deviation.

Within-group differences in EG Group are shown in Table 2. In the EG group (n = 11), statistically significant differences were found in terms of UPDRS-III, BBT, TUG, 6MWT, MoCA, PDQ-39, Stroop TD, visual delayed recall, visual recognition, VMPT immediate recall, and BNT (p < 0.05).

**Table 2:** Within group findings in EG group.

EG group	Pre-treatment	Post-treatment	Mean difference	Confidence of interval (lower to upper)	F	Effect size (Cohen’s d)	p value
	Avg ± SD	Avg ± SD					
**Motor findings**							
UPDRS	13.63 ± 7.44	6.90 ± 3.56	6.727	2.902 to 10.552	15.356	0.606	0.003*
BBT	44.63 ± 9.67	51.27 ± 5.93	− 6.636	− 9.791 to − 3.481	21.966	0.687	0.001*
TUG	14.90 ± 2.86	13.01 ± 2.12	1.886	0.590 to 3.182	10.519	0.513	0.009*
6MWT	198.90 ± 56.26	283.63 ± 69.06	− 84.727	− 125.893 to − 43.561	21.031	0.678	0.001*
ABCS	995.90 ± 351.10	1217.27 ± 352.13	− 221.364	− 476.324 to 33.597	3.742	0.272	0.082
**Cognition and mood**							
MMSE	26.36 ± 1.85	26.63 ± 1.62	− 0.273	− 1.515 to 0.969	0.239	0.023	0.635
MoCA	22.27 ± 2.19	24.54 ± 1.50	− 2.273	− 3.127 to − 1.418	35.112	0.778	0.000*
GDS	7.09 ± 6.13	4.90 ± 3.23	2.182	− 1.281 to 5.644	1.971	0.165	0.191
PDQ-39	42.72 ± 26.38	24.72 ± 15.81	18.000	3.819 to 32.181	7.998	0.444	0.018*
**Attention**							
DS forward	5.27 ± 0.90	5.09 ± 1.04	0.182	− 0.478 to 0.841	0.377	0.036	0.553
DS backward	3.45 ± 1.21	3.36 ± 0.92	0.091	− 0.468 to 0.649	0.132	0.013	0.724
**Executive functions**							
Stroop TD	71.95 ± 27.96	57.94 ± 23.81	14.011	1.115 to 26.907	5.860	0.369	0.036*
VF–fruit name pairs	7.00 ± 0.89	6.98 ± 1.67	0.015	− 0.936 to 0.966	0.001	0.000	0.972
Categorical fluency	15.81 ± 5.94	17.00 ± 6.61	− 1.182	− 3.981 to 1.168	0.885	0.081	0.369
Phonemic fluency	23.81 ± 14.55	24.00 ± 6.38	− 0.182	− 6.952 to 6.589	0.004	0.000	0.953
Abstract thinking	2.72 ± 0.64	2.63 ± 1.02	0.091	− 0.380 to 0.562	0.185	0.018	0.676
Similarities	9.18 ± 1.07	9.36 ± 1.20	− 0.182	− 0.966 to 0.603	0.267	0.026	0.617
CDT	2.27 ± 0.64	3.18 ± 0.98	− 0.909	− 1.926 to 0.108	3.968	0.284	0.074
**Memory**							
Visual immediate recall	5.45 ± 3.35	7.36 ± 3.29	− 1.909	− 4.043 to 0.225	3.973	0.284	0.074
Visual delayed recall	3.09 ± 2.21	7.00 ± 3.68	− 3.909	− 6.022 to − 1.796	16.994	0.630	0.002*
Visual recognition	1.81 ± 0.87	3.09 ± 0.70	− 1.273	− 2.073 to − 0.473	12.564	0.557	0.005*
Logical immediate recall	14.63 ± 4.52	13.07 ± 3.51	1.558	− 1.818 to 4.934	1.057	0.096	0.328
Logical delayed recall	14.36 ± 4.94	12.51 ± 4.03	1.844	− 1.617 to 5.306	1.409	0.124	0.263
VMPT immediate recall	3.00 ± 1.18	4.18 ± 1.25	− 1.182	− 2.215 to − 0.149	6.500	0.394	0.029*
VMPT delayed recall	7.09 ± 2.50	6.81 ± 3.99	0.273	− 2.531 to 3.077	0.047	0.005	0.833
VMPT recognition	4.18 ± 1.60	4.27 ± 2.28	− 0.545	− 2.088 to 0.997	0.621	0.058	0.449
VMPT total	11.36 ± 2.20	11.54 ± 3.77	− 0.182	− 2.849 to 2.485	0.023	0.002	0.882
**Language**							
BNT	22.81 ± 4.06	25.90 ± 3.75	− 3.091	− 5.049 to − 1.133	12.377	0.553	0.006*
**Visual spatial functions**							
BFR	42.72 ± 4.10	41.27 ± 2.14	1.455	− 0.699 to 3.608	2.265	0.185	0.163
Line orientation	42.08 ± 10.44	48.79 ± 5.93	− 6.711	− 14.316 to 0.895	3.865	0.279	0.078

*Avg* average, *SD* standard deviation, *UPDRS* Unified Parkinson’s Disease Rating Scale, *BBT* Berg Balance Test, *TUG* Timed-up and Go Test, *6MWT* 6 min walk test, *ABCS* Activities-specific Balance Confidence Scale, *MMSE* Mini-Mental State Examination, *MoCA* Montreal Cognitive Assessment, *GDS* Geriatric Depression Scale, *PDQ-39* Parkinson's Disease Questionnaire, *TD* Time Difference, *VF* Verbal Fluency, *CDT* Clock Drawing Test, *VMPT* Verbal Memory Process Test, *BNT* Boston Naming Test, *BFR* Benton Face Recognition. *p < 0.05.

Within-group differences in ET Group are shown in Table 3. There were statistically significant differences in terms of UPDRS-III, BBT, TUG, 6MWT, VF-fruit name pairs, similarities, visual recognition, and VMPT delayed recall in the ET Group (n = 13) (p < 0.05).

**Table 3:** Within group findings in ET group.

ET group	Pre-treatment	Post-treatment	Mean difference	Confidence of interval (lower to upper)	F	Effect size (Cohen’s d)	p value
	Avg ± SD	Avg ± SD					
**Motor findings**							
UPDRS	15.30 ± 8.83	9.53 ± 9.16	5.769	1.813 to 9.725	10.096	0.457	0.008*
BBT	44.23 ± 5.89	53.30 ± 2.46	− 9.077	− 11.840 to − 6.314	51.223	0.810	0.000*
TUG	16.05 ± 6.95	12.61 ± 5.44	3.444	0.227 to 6.660	5.442	0.312	0.038*
6MWT	195.15 ± 75.36	242.76 ± 76.35	− 47.615	− 88.199 to − 7.032	6.535	0.353	0.025*
ABCS	1033.46 ± 335.29	1045.38 ± 402.31	− 11.923	− 193.729 to 169.883	0.020	0.002	0.889
**Cognition and mood**							
MMSE	25.15 ± 2.79	24.84 ± 3.50	0.308	− 1.221 to 1.836	0.192	0.016	0.669
MoCA	22.76 ± 3.39	23.00 ± 4.91	− 0.231	− 1.907 to 1.445	0.090	0.007	0.769
GDS	10.46 ± 8.56	9.00 ± 8.55	1.462	− 2.357 to 5.280	0.695	0.055	0.421
PDQ-39	48.84 ± 24.82	33.00 ± 27.53	15.846	− 1.668 to 33.360	3.886	0.245	0.072
**Attention**							
DS forward	5.57 ± 1.11	5.15 ± 0.80	0.417	− 0.104 to 0.938	3.037	0.202	0.107
DS backward	3.41 ± 0.86	3.23 ± 0.83	0.186	− 0.390 to 0.762	0.495	0.040	0.495
**Executive functions**							
Stroop TD	68.25 ± 22.21	65.50 ± 22.16	2.756	− 9.945 to 15.457	0.223	0.018	0.645
VF–fruit name pairs	5.76 ± 1.92	6.92 ± 1.93	− 1.154	− 2.166 to − 0.141	6.164	0.339	0.029*
Categorical fluency	15.15 ± 4.21	16.07 ± 3.81	− 0.923	− 2.934 to 1.088	1.000	0.077	0.337
Phonemic fluency	22.09 ± 12.89	24.56 ± 7.68	− 2.471	− 9.800 to 4.857	0.540	0.043	0.477
Abstract thinking	2.66 ± 0.62	2.61 ± 0.65	0.054	− 0.409 to 0.518	0.066	0.005	0.802
Similarities	7.72 ± 2.06	8.92 ± 1.32	− 1.199	− 2.555 to 0.142	6.115	0.338	0.029*
CDT	3.00 ± 1.52	2.67 ± 1.49	0.324	− 0.685 to 1.322	0.489	0.039	0.498
**Memory**							
Visual immediate recall	6.61 ± 4.03	8.37 ± 5.83	− 1.763	− 4.637 to 1.112	1.784	0.129	0.206
Visual delayed recall	6.07 ± 4.62	7.00 ± 4.83	− 0.923	− 2.531 to 0.684	1.565	0.115	0.235
Visual recognition	1.84 ± 1.67	2.84 ± 1.40	− 0.997	− 1.981 to − 0.013	4.869	0.289	0.048*
Logical immediate recall	12.47 ± 3.61	10.83 ± 4.48	1.636	− 0.839 to 4.111	2.073	0.147	0.175
Logical delayed recall	12.36 ± 5.13	11.29 ± 5.66	1.065	− 2.478 to 4.607	0.429	0.034	0.525
VMPT immediate recall	4.23 ± 1.69	4.00 ± 1.35	0.231	− 1.007 to 1.468	0.165	0.014	0.692
VMPT delayed recall	5.53 ± 3.33	8.07 ± 4.29	− 2.538	− 4.411 to − 0.666	8.724	0.421	0.012*
VMPT recognition	4.15 ± 3.67	3.38 ± 2.02	0.769	− 1.832 to 3.370	0.415	0.033	0.531
VMPT Total	9.69 ± 4.66	11.46 ± 4.23	− 1.769	− 3.992 to 0.453	3.009	0.200	0.108
**Language**							
BNT	25.39 ± 4.76	25.92 ± 5.20	− 0.526	− 1.719 to 0.667	0.922	0.071	0.356
**Visual spatial functions**							
BFR	40.92 ± 3.66	40.07 ± 5.00	0.846	− 1.140 to 2.833	0.861	0.067	0.372
Line orientation	42.76 ± 13.94	44.97 ± 11.13	− 3.217	− 9.289 to 2.856	1.332	0.100	0.271

*Avg* average, *SD* standard deviation, *UPDRS* Unified Parkinson’s Disease Rating Scale, *BBT* Berg Balance Test, *TUG* Timed-up and Go Test, *6MWT* 6 min walk test, *ABCS* Activities-specific Balance Confidence Scale, *MMSE* Mini-Mental State Examination, *MoCA* Montreal Cognitive Assessment, *GDS* Geriatric Depression Scale, *PDQ-39* Parkinson's Disease Questionnaire, *TD* Time Difference, *VF* Verbal Fluency, *CDT* Clock Drawing Test, *VMPT* Verbal Memory Process Test, *BNT* Boston Naming Test, *BFR* Benton Face Recognition. *p < 0.05.

Between group differences are shown in Table 4. At the baseline, there was a statistically significant difference in VMPT immediate recall between groups (p < 0.05). In the time × group interactions, there were statistically significant differences in MoCA, Visual delayed recall, and BNT in favor of EG Group (p < 0.05).

**Table 4:** Between group findings.

	Pre-treatment			Post-treatment			Difference			
	EG group	ET group	p value	EG group	ET group	p value	MD (CI)	F	Effect size (d)	p value
	Avg ± SD	Avg ± SD		Avg ± SD	Avg ± SD					
**Motor findings**										
UPDRS	13.63 ± 7.44	15.30 ± 8.83	0.839	6.90 ± 3.56	9.53 ± 9.16	0.771	− 2.150 (− 8.166 to 3.866)	0.143	0.006	0.708
BBT	44.63 ± 9.67	44.23 ± 5.89	0.597	51.27 ± 5.93	53.30 ± 2.46	0.555	− 0.815 (− 5.844 to 4.215)	1.656	0.070	0.212
TUG	14.90 ± 2.86	16.05 ± 6.95	0.862	13.01 ± 2.12	12.61 ± 5.44	0.622	− 0.378 (− 4.167 to 3.411)	0.843	0.037	0.368
6MWT	198.90 ± 56.26	195.15 ± 75.36	0.816	283.63 ± 69.06	242.76 ± 76.35	0.283	22.311 (− 30.753 to 75.375)	1.969	0.082	0.174
ABCS	995.90 ± 351.10	1033.46 ± 335.29	0.839	1217.27 ± 352.13	1045.38 ± 402.31	0.385	67.168 (− 204.566 to 338.902)	2.276	0.094	0.146
**Cognition and mood**										
MMSE	26.36 ± 1.85	25.15 ± 2.79	0.292	26.63 ± 1.62	24.84 ± 3.50	0.291	1.500 (− 0.513 to 3.513)	0.398	0.018	0.535
MoCA	22.27 ± 2.19	22.76 ± 3.39	0.430	24.54 ± 1.50	23.00 ± 4.91	0.726	0.524 (− 2.177 to 3.226)	5.308	0.186	0.035*
GDS	7.09 ± 6.13	10.46 ± 8.56	0.352	4.90 ± 3.23	9.00 ± 8.55	0.293	− 3.731 (− 9.267 to 1.806)	0.091	0.004	0.765
PDQ-39	42.72 ± 26.38	48.84 ± 24.82	0.562	24.72 ± 15.81	33.00 ± 27.53	0.772	− 7.196 (− 24.706 to 10.315)	0.042	0.002	0.840
**Attention**										
DS forward	5.27 ± 0.90	5.57 ± 1.11	0.437	5.09 ± 1.04	5.15 ± 0.80	1.000	− 0.180 (− 0.910 to 0.549)	0.390	0.017	0.539
DS backward	3.45 ± 1.21	3.41 ± 0.86	1.000	3.36 ± 0.92	3.23 ± 0.83	0.665	0.085 (− 0.635 to 0.805)	0.660	0.003	0.799
**Executive functions**										
Stroop TD	71.95 ± 27.96	68.25 ± 22.21	0.931	57.94 ± 23.81	65.50 ± 22.16	0.706	− 1.931 (− 20.410 to 16.549)	1.848	0.077	0.188
VF–fruit name pairs	7.00 ± 0.89	5.76 ± 1.92	0.065	6.98 ± 1.67	6.92 ± 1.93	0.552	0.646 (− 0.624 to 1.917)	3.335	0.132	0.081
Categorical fluency	15.81 ± 5.94	15.15 ± 4.21	0.907	17.00 ± 6.61	16.07 ± 3.81	0.954	0.794 (− 3.311 to 4.898)	0.029	0.001	0.867
Phonemic fluency	23.81 ± 14.55	22.09 ± 12.89	0.505	24.00 ± 6.38	24.56 ± 7.68	0.908	0.581 (− 7.357 to 8.518)	0.247	0.011	0.624
Abstract thinking	2.72 ± 0.64	2.66 ± 0.62	0.567	2.63 ± 1.02	2.61 ± 0.65	0.677	0.039 (− 0.512 to 0.590)	0.015	0.001	0.905
Similarities	9.18 ± 1.07	7.72 ± 2.06	0.059	9.36 ± 1.20	8.92 ± 1.32	0.323	0.949 (− 0.146 to 2.044)	2.695	0.109	0.115
CDT	2.27 ± 0.64	3.00 ± 1.52	0.167	3.18 ± 0.98	2.67 ± 1.49	0.480	− 0.111 (− 1.089 to 0.867)	3.536	0.138	0.073
**Memory**										
Visual immediate recall	5.45 ± 3.35	6.61 ± 4.03	0.466	7.36 ± 3.29	8.37 ± 5.83	0.884	− 1.088 (− 4.326 to 2.151)	0.008	0.000	0.932
Visual delayed recall	3.09 ± 2.21	6.07 ± 4.62	0.143	7.00 ± 3.68	7.00 ± 4.83	0.907	− 1.493 (− 4.708 to 1.722)	6.358	0.224	0.019*
Visual recognition	1.81 ± 0.87	1.84 ± 1.67	0.881	3.09 ± 0.70	2.84 ± 1.40	0.927	0.110 (− 0.767 to 0.987)	0.217	0.010	0.646
Logical immediate recall	14.63 ± 4.52	12.47 ± 3.61	0.139	13.07 ± 3.51	10.83 ± 4.48	0.163	2.204 (− 0.658 to 5.066)	0.002	0.000	0.967
Logical delayed recall	14.36 ± 4.94	12.36 ± 5.13	0.296	12.51 ± 4.03	11.29 ± 5.66	0.560	1.613 (− 1.940 to 5.165)	0.118	0.005	0.735
VMPT immediate recall	3.00 ± 1.18	4.23 ± 1.69	0.017*	4.18 ± 1.25	4.00 ± 1.35	0.633	− 0.524 (− 1.421 to 0.372)	3.537	0.139	0.073
VMPT delayed recall	7.09 ± 2.50	5.53 ± 3.33	0.254	6.81 ± 3.99	8.07 ± 4.29	0.337	0.147 (− 2.515 to 2.809)	3.579	0.140	0.072
VMPT recognition	4.18 ± 1.60	4.15 ± 3.67	0.539	4.27 ± 2.28	3.38 ± 2.02	0.113	0.685 (− 0.889 to 2.260)	0.824	0.036	0.374
VMPT Total	11.36 ± 2.20	9.69 ± 4.66	0.641	11.54 ± 3.77	11.46 ± 4.23	0.883	0.878 (− 2.007 to 3.762)	1.032	0.045	0.321
**Language**										
BNT	22.81 ± 4.06	25.39 ± 4.76	0.115	25.90 ± 3.75	25.92 ± 5.20	0.705	− 1.297 (− 5.005 to 2.412)	6.551	0.229	0.018*
**Visual spatial functions**										
BFR	42.72 ± 4.10	40.92 ± 3.66	0.321	41.27 ± 2.14	40.07 ± 5.00	0.540	1.500 (− 1.531 to 4.531)	0.209	0.009	0.652
Line orientation	42.08 ± 10.44	41.76 ± 13.94	0.727	48.79 ± 5.93	44.97 ± 11.13	0.523	2.067 (− 5.968 to 10.103)	0.642	0.028	0.432

*Avg* average, *SD* standard deviation, *UPDRS* Unified Parkinson’s Disease Rating Scale, *BBT* Berg Balance Test, *TUG* Timed-up and Go Test, *6MWT* 6 min walk test, *ABCS* Activities-specific Balance Confidence Scale, *MMSE* Mini-Mental State Examination, *MoCA* Montreal Cognitive Assessment, *GDS* Geriatric Depression Scale, *PDQ-39* Parkinson's Disease Questionnaire, *TD* Time Difference, *VF* Verbal Fluency, *CDT* Clock Drawing Test, *VMPT* Verbal Memory Process Test, *BNT* Boston Naming Test, *BFR* Benton Face Recognition. *p < 0.05.

### Neuroimaging results

Before treatment comparisons showed no significant differences between groups in terms of cerebral activities. Our GLM in the rs-fMRI analysis needed all patients with complete data (pre and post). rs-fMRI data analysis was completed with 8 patients in the EG group and 10 patients in the ET group caused by the dropouts in the neuroimaging data due to incompatibilities in the post treatment data.

**Figure 2:**
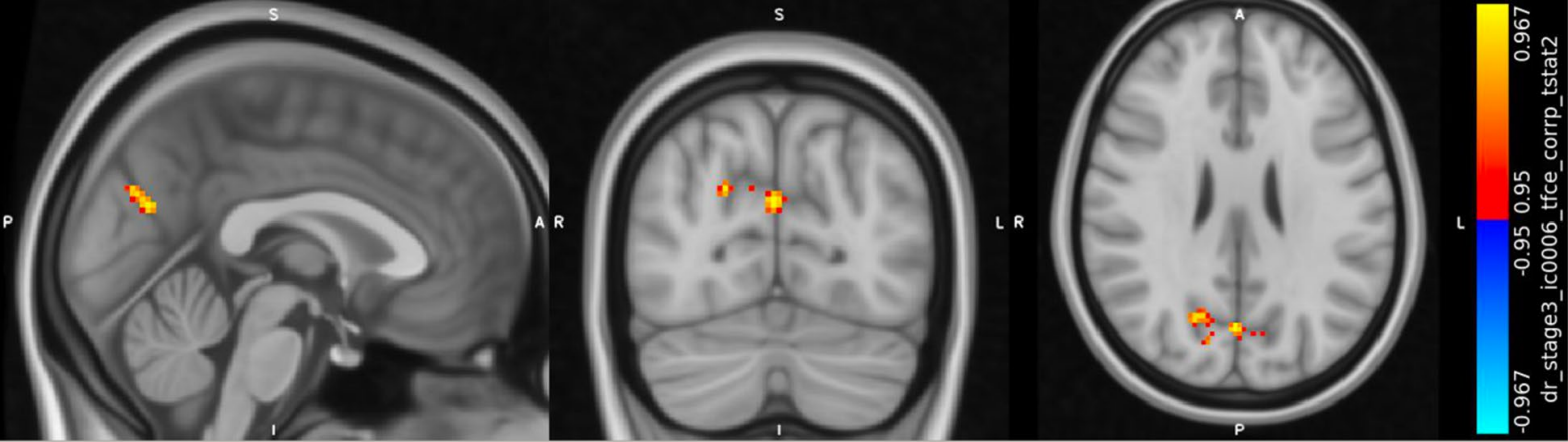
Results of rs-fMRI analysis in the between group comparison. Between groups comparisons, results of Dual Regression with group × time interaction, showing increased activation in the precuneus cortex (Harvard-Oxford Atlas), corrected for multiple comparisons with threshold-free cluster enhancement (tfce, p-value < 0.05). Peak MNI152 coordinate: [0 −72 25]; Number of voxels in the significant cluster: 154; Minimum p-value: 0.0326; t-value: 4.75

Between group comparisons showed increased activation in the precuneus cortex (Harvard–Oxford Atlas) after treatment in the favor of the EG group (p < 0.05). Results of Dual Regression were corrected for multiple comparisons with threshold-free cluster enhancement (tfce, p-value < 0.05). The result of between group comparison in shown in Fig. 2.

## Discussion

The results of this study showed the beneficial effects of Virtual Reality-Based EG in patients with PD compared to conventional physical therapy. Both EG and ET improved the patients’ clinical outcomes in terms of motor aspect of the disease, balance, and gait. Importantly, only EG was effective in significantly improving patients’ QoL and general cognitive status (MoCA). Neuropsychological assessment revealed improvement in the EG group in terms of Stroop TD, visual delayed recall, visual recognition, and VMPT immediate recall. Additionally, ET led to significant improvements in VF-fruit name pairs, similarities, visual recognition, and VMPT delayed recall after the intervention. Importantly, between group comparisons considering the interaction of group × time effect, showed superiority of EG in terms of MoCA, delayed visual recall memory, and BNT. These results were consistent in the within-group and between-group analysis. Finally, rs-fMRI analysis showed increasing activity in the precuneus region in the time × group interaction meaning that considering two groups’ data before and after the intervention (totally 4 levels in the GLM) EG led to increased activity in the precuneus compared to the ET. No between group interaction effect was seen in the favor of the ET group in rs-fMRI analysis.

DMN is involved in cognitive decline in PD, and patients with cognitive impairment demonstrated decreased FC in nodes of DMN^16,47^. While specific coupling/decoupling patterns are crucial in cognitive behaviors, loss of normal anticorrelation between Dorsal Attention Network and DMN is also reported in PD patients with MCI (PD-MCI) compared to healthy controls^48^. The most consistent result that may help to distinguish PD patients with and without cognitive impairment is decreased FC within the DMN^47^, which was initially proposed in a research using a small sample of cognitively unimpaired PD patients suing ICA^49^. Later it has been reported that functional disconnection of posterior brain areas can occur before clinically detectable cognitive impairment in PD^49^. Deteriorated FC in posterior cortical areas has been linked to the development of cognitive impairment over 3 years in a longitudinal fMRI research^50^. A study investigating the FC between DMN and other brain regions, chose precuneus and medial prefrontal cortex as DMN hub regions. The result showed that precuneus exhibited decreased FC with nodes of motor system such as basal ganglia, motor cortex and thalamus in patients with PD. The results of this study imply that besides impairments in the motor system in PD, connectivity of motor networks to DMN is also impaired^51^. We believe that the results of our study can contribute to this context. The increased precuneus activity after intervention can be accepted as an indicator of increased connectivity inside DMN and therefore help to normalize between-network connections of DMN with other networks and therefore help to improve cognitive functions. Consistent significance of MOCA in the EG group can admit this claim. Patients with PD, after performing an EG program, which is a combination of motor and cognitive tasks, can exhibit increased FC in the cognitive networks which in future might help to improve the motor networks as well. In other words, interventions like EG and VR, may be able to reverse the prognoses of the diseases by affecting the FC in the cognitive networks and further have an impact on the motor systems connectivity. The short period of intervention in our protocol (4 weeks, 12 sessions) may be the reason that we did not see functional changes in the motor networks.

Precuneus has a heterogeneous structure and complex FC. Precuneus and the posterior cingulate cortex appear to act in conjunction in terms of function and cognition. Precuneus/posterior cingulate cortex are shown to be part of DMN and have strong interaction between other regions of DMN and other networks as well^52^. In a recent study, Arterial Spin Labeling (ASL) has been used for a quantitative measurement of cerebral blood flow and link how the altered neurovascular perfusion in PD may interact with the disrupted FC. It has been hypothesized that PD-MCI patients may exhibit hypoperfusion in the parietal-occipital network, and the results have confirmed that PD-MCI patients show hypoperfusion in the parietal memory network in the precuneus. Moreover, decreased Precuneus-FC in the right striatum was shown, which is commented to be a result of structural and functional connection between the precuneus and striatum. Importantly, decreased perfusion and disrupted FC in the precuneus has been positively associated with memory performance in these patients. This study suggests the important role of precuneus in memory deficits in PD patients^53^. More studies additionally have shown that precuneus is one of the main nodes of the parietal memory network (PMN). This network is active during activities involving memory encoding and retrieval^54,55^. Notably, precuneus has been linked to visuo-spatial integration, memory, and self-awareness. Functional neuroimaging data consistently relate the precuneus to self-consciousness in this way. On the other hand, the posterior cingulate cortex is also important in episodic memory, particularly in recalling self-related events^56^. This region is also important for monitoring one's own movement in space^56^. Reduced connection between the posterior cingulate and bilateral medial temporal lobe has been identified as altered FC in the DMN. Furthermore, decreased FC in DMN are shown to be associated with worse verbal and visual memory and visual skills in PD^57,58^. Increased FC in the precuneus after the intervention in the EG group in our study, and consistent significance in the visual memory and verbal task can be assumed to be correlated in this context. We believe that the EG tasks, which provide visual and auditory feedback to the patients, motivate patients to remember the activities for better performance, while they try to imagine themselves in the role of the avatar and organize the internally represented visual images, can be an explanation of the significance of Precuneus activation, visual memory tasks and verbal skills in the EG group. In addition, EG sessions include observing the actions and following the game rules to complete the sessions. Therefore, patients need to use their recognition capability to keep up with the tasks. The nature of the EG tasks may have been effective in modulating this “recognition ability” and improved the BNT in the EG group consistently. Although between-group analysis did not show significant improvement in the experimental group compared to the control group, QoL improved in both groups, and the improvement in the ET group was almost significant. We believe that the nature of the individualized exercises in both groups could have had a positive effect on patients’ QoL. Patients in both groups were in continuous communication with the Physical Therapist and were able to consult all the issues of their life to find a solution. Interventions in the ET group needed more communication between Physical Therapist and the patients, and this may have been effective in improving their QoL effectively. However, while both interventions were effective in this context, they were not superior to each other in improving patients’ QoL.

Finally, our study may also be in line with the dual syndrome hypothesis. This theory implies that dysfunctions in PD can be due to dopamine depletion or cholinergic dysfunction. Based on the hypothesis, Dopamine depletion can be present in the frontostriatal regions (anterior frontostriatal executive syndrome) and can be seen in PD-MCI patients with cognitive problems such as executive functions and working memory. On the other hand, cholinergic dysfunction can be seen in the posterior cortical/parietal regions and PD patients in this subgroup can demonstrate visuo-spatial dysfunction and semantic fluency impairment. Therefore, visuospatial and perception problems in PD can be more related to the posterior system, rather than the dopamine depletion^57,59^. Considering the rs-fMRI and clinical results it can be inferred from our result that interventions like EG can be more successful in affecting the posterior system in PD. EG provides visual inputs to the participants and may have been helpful in improving memory secondarily by recruiting regions in the posterior system and PMN, including precuneus. However, long term effects of EG on brain FC in PD patients should be further investigated.

Regarding the motor symptoms, a recent meta-analysis study has reviewed all the studies that utilized EG in PD^60^. Results have shown that EG can be more beneficial in improving QoL (PDQ-39), and balance (BBS) compared to the active exercises. Thus, functional mobility (TUG) is reported to be the same in both interventions. Our results are partially admitting these results, as improvements in the BBS and TUG were seen in both groups, and there was only improvement in the PDQ-39 in the EG group. However, improvement in the QoL was not consistent in the between group comparison. We believe that in terms of motor outcomes, EG and ET can exhibit the similar effects, which means EG can be an appropriate interactive alternative to the conventional physiotherapy approaches, although EG can have beneficiary additional effects in terms of QoL and general cognition. Therefore, even in short periods of rehabilitation application in patients with PD, patients can benefit from EG to improve cognitive functions and QoL, beside the effects on motor symptoms. Additionally, our results may represent clinically important differences (CIDs) for UPDRS-III. Both interventions were helpful in improving CIDs for UPDRS-III which implies their similarity in improving motor symptoms in PD patients^61^. However, it is hard to comment which of the interventions were more effective in this field. Significance in the precuneus and the improvements in the UPDRS-III can logically be linked with increased connectivity of the DMN with the motor networks.

Our study has some limitations. First, the sample size in our study was small. Many patients rejected to participate in two MRI scans. Of the participants who accepted to participate in the study, some did not attend the second evaluations due to their unwillingness to enter the scanner for the second time. Second, the duration of our intervention was short (4 weeks, 12 sessions). Although there are some studies in the literature with 5/6 weeks of intervention, the total number of interventions in these studies are similar to ours. We are aware that applying longer intervention periods could be better but considering the municipality transportation problems and long distances to the hospital, we assumed that longer periods (more the 4 weeks) would increase our drop-outs due the patients unwillingness to attend the treatments till the end date and participate in the post-treatment evaluations. Third, we did not subgrouped our patients in terms of cognitive impairment and depression. Therefore, our study shows the effects of interventions on the pre-existing cognition. Further studies comparing the effects of VR based EG on different PD subgroups are needed. Fourth, considering the learning effects in the cognitive tests, we used A/B forms of VMPT and Logical Recall Test. However, for the other cognitive tests we did not used parallel versions. While learning effects are mostly dominant in memory tests, we have used B forms for two of the tests. Therefore, parallel versions of the cognitive test should be considered in further studies.

In conclusion, EG can be an effective alternative in terms of motor and cognitive outcomes in patients with PD. EG may have a positive effect in normalizing altered brain FC which consequently can have beneficial effects on patients’ cognitive functions and motor symptoms. Whenever possible, using EG and ET in combination, may have the better effects on patients daily living and patients can benefit from the advantages of both interventions. However, further studies using larger samples, longer treatment periods and follow-up evaluations are warranted to better determine the long-term effects of VR based EG in patients with PD.

## References

[1] Connolly BS, Lang AE (2014). Pharmacological treatment of Parkinson disease: A review. JAMA J. Am. Med. Assoc..

[2] Bloem BR, de Vries NM, Ebersbach G (2015). Nonpharmacological treatments for patients with Parkinson’s disease. Mov. Disord..

[3] Schaeffer E (2019). Effects of exergaming on attentional deficits and dual-tasking in Parkinson’s disease. Front. Neurol..

[4] Chen Y, Gao Q, He CQ, Bian R (2020). Effect of virtual reality on balance in individuals with Parkinson disease: A systematic review and meta-analysis of randomized controlled trials. Phys. Ther..

[5] Dockx, K. *et al.* Virtual reality for rehabilitation in Parkinson’s disease. *Cochrane Database Syst. Rev.* **2016** (2016). 10.1002/14651858.CD010760.pub2 PMC6463967 28000926

[6] Pompeu JE (2012). Effect of Nintendo Wii™ based motor and cognitive training on activities of daily living in patients with Parkinson’s disease: A randomised clinical trial. Physiother. (United Kingdom).

[7] Cerasa A (2014). Neurofunctional correlates of attention rehabilitation in Parkinson’s disease: An explorative study. Neurol. Sci..

[8] Díez-Cirarda M (2017). Increased brain connectivity and activation after cognitive rehabilitation in Parkinson’s disease: A randomized controlled trial. Brain Imaging Behav..

[9] Zimmermann R (2014). Cognitive training in Parkinson disease: Cognition-specific vs nonspecific computer training. Neurology.

[10] Maidan I (2017). Disparate effects of training on brain activation in Parkinson disease. Neurology.

[11] Huang C (2008). Metabolic abnormalities associated with mild cognitive impairment in Parkinson disease. Neurology.

[12] Yong SW, Yoon JK, An YS, Lee PH (2007). A comparison of cerebral glucose metabolism in Parkinson’s disease, Parkinson’s disease dementia and dementia with Lewy bodies. Eur. J. Neurol..

[13] Gruszka A, Hampshire A, Barker RA, Owen AM (2017). Normal aging and Parkinson’s disease are associated with the functional decline of distinct frontal-striatal circuits. Cortex.

[14] Gorges M (2015). To rise and to fall: Functional connectivity in cognitively normal and cognitively impaired patients with Parkinson’s disease. Neurobiol. Aging.

[15] Prell T (2018). Structural and functional brain patterns of non-motor syndromes in Parkinson’s disease. Front. Neurol..

[16] Tahmasian M (2017). Resting-state functional reorganization in Parkinson’s disease: An activation likelihood estimation meta-analysis. Cortex.

[17] Ruppert MC (2021). The default mode network and cognition in Parkinson’s disease: A multimodal resting-state network approach. Hum. Brain Mapp..

[18] Filippi M, Elisabetta S, Piramide N, Agosta F (2018). Functional MRI in idiopathic Parkinson’s disease. Int. Rev. Neurobiol..

[19] Barbour V (2017). CONSORT Statement for randomized Trials of nonpharmacologic treatments: A 2017 update and a CONSORT extension for nonpharmacologic Trial Abstracts. Ann. Intern. Med..

[20] Fénelon G, Mahieux F, Huon R, Ziégler M (2000). Hallucinations in Parkinson’s disease. Prevalence, phenomenology and risk factors. Brain.

[21] Gelb DJ, Oliver E, Gilman S (1999). Diagnostic criteria for Parkinson disease. Arch. Neurol..

[22] Goetz CG (2004). Movement Disorder Society Task Force report on the Hoehn and Yahr staging scale: Status and recommendations. Mov. Disord..

[23] Liao YY (2015). Virtual reality-based training to improve obstacle-crossing performance and dynamic balance in patients with Parkinson’s disease. Neurorehabil. Neural Repair.

[24] Alves MLM (2018). Nintendo Wii™ versus Xbox Kinect™ for assisting people with Parkinson’s disease. Percept. Mot. Skills.

[25] Santos P, Machado T, Santos L, Ribeiro N, Melo A (2019). Efficacy of the Nintendo Wii combination with Conventional Exercises in the rehabilitation of individuals with Parkinson’s disease: A randomized clinical trial. NeuroRehabilitation.

[26] Akbostanci MC (2018). Turkish standardization of movement disorders society unified Parkinson’s Disease Rating Scale and Unified Dyskinesia Rating Scale. Mov. Disord. Clin. Pract..

[27] La Porta F (2012). Is the berg balance scale an internally valid and reliable measure of balance across different etiologies in neurorehabilitation? A revisited Rasch analysis study. Arch. Phys. Med. Rehabil..

[28] Lohnes CA, Earhart GM (2010). External validation of abbreviated versions of the activities-specific balance confidence scale in Parkinson’s disease. Mov. Disord..

[29] Nocera JR (2013). Using the timed up & go test in a clinical setting to predict falling in Parkinson’s disease. Arch. Phys. Med. Rehabil..

[30] Falvo MJ, Earhart GM (2009). Reference equation for 6-minute walk in individuals with Parkinson disease. J. Rehabil. Res. Dev..

[31] Ozdilek B, Kenangil G (2014). Validation of the Turkish version of the Montreal cognitive assessment scale (MoCA-TR) in patients with Parkinsons disease. Clin. Neuropsychol..

[32] Tanör, Ö. Ö. Öktem sözel bellek süreçleri testi. (Öktem-SBST) el kitabı (2011).

[33] Elwood RW (1991). The Wechsler Memory Scale-Revised: Psychometric characteristics and clinical application. Neuropsychol. Rev..

[34] Sisco SM, Slonena E, Okun MS, Bowers D, Price CC (2016). Parkinson’s disease and the Stroop color word test: Processing speed and interference algorithms. Clin. Neuropsychol..

[35] Crawford, J. R., Parker, D. M. & McKinlay, W. W. *A Handbook of Neuropsychological Assessment*. 10.4324/9780429490316 (2018).

[36] Karakaş, S. Bilnot—Yetişkin (2 Cilt Takım)—Sirel Karakaş—Google Books. https://books.google.com.tr/books?hl=en&lr=&id=cMOiDwAAQBAJ&oi=fnd&pg=PA255&dq=Bilnot+-+Yetişkin+(2+Cilt+Takım)+,+Elvin+Doğutepe+Dinçer+&ots=dUgwni1gOM&sig=PtPYWBIY2CjuN-RbR2urlE9Nrww&redir_esc=y#v=onepage&q=Bilnot-Yetişkin(2CiltTakım)%2CElvinDoğu.

[37] Warden C, Hwang J, Marshall A, Fenesy M, Poston KL (2016). The effects of dopamine on digit span in Parkinson’s disease. J. Clin. Mov. Disord..

[38] Ekinci Soylu A, Cangöz B (2018). Adaptation and norm determination study of the Boston naming test for healthy Turkish elderly. Noropsikiyatri Ars..

[39] Lopez FV (2018). Does the Geriatric Depression Scale measure depression in Parkinson’s disease?. Int. J. Geriatr. Psychiatry.

[40] Hagell P, Nygren C (2007). The 39 item Parkinson’s disease questionnaire (PDQ-39) revisited: Implications for evidence based medicine. J. Neurol. Neurosurg. Psychiatry.

[41] van Dijk KRA, Sabuncu MR, Buckner RL (2012). The influence of head motion on intrinsic functional connectivity MRI. Neuroimage.

[42] Jenkinson M, Bannister P, Brady M, Smith S (2002). Improved optimization for the robust and accurate linear registration and motion correction of brain images. Neuroimage.

[43] Griffanti L (2017). Hand classification of fMRI ICA noise components. Neuroimage.

[44] Smith SM (2002). Fast robust automated brain extraction. Hum. Brain Mapp..

[45] Tomlinson CL (2010). Systematic review of levodopa dose equivalency reporting in Parkinson’s disease. Mov. Disord..

[46] Shirer WR, Ryali S, Rykhlevskaia E, Menon V, Greicius MD (2012). Decoding subject-driven cognitive states with whole-brain connectivity patterns. Cereb. Cortex.

[47] Wolters AF (2019). Resting-state fMRI in Parkinson’s disease patients with cognitive impairment: A meta-analysis. Parkinson Relat. Disord..

[48] Baggio HC (2015). Cognitive impairment and resting-state network connectivity in Parkinson’s disease. Hum. Brain Mapp..

[49] Tessitore A, Cirillo M, De Micco R (2019). Functional connectivity signatures of Parkinson’s disease. J. Parkinsons Dis..

[50] Dubbelink KTEO (2014). Functional connectivity and cognitive decline over 3 years in Parkinson disease. Neurology.

[51] Thibes RB (2017). Altered functional connectivity between precuneus and motor systems in Parkinson’s disease patients. Brain Connect..

[52] Fransson P, Marrelec G (2008). The precuneus/posterior cingulate cortex plays a pivotal role in the default mode network: Evidence from a partial correlation network analysis. Neuroimage.

[53] Jia X, Li Y, Li K, Liang P, Fu X (2019). Precuneus dysfunction in Parkinson’s disease with mild cognitive impairment. Front. Aging Neurosci..

[54] Gilmore AW, Nelson SM, McDermott KB (2015). A parietal memory network revealed by multiple MRI methods. Trends Cogn. Sci..

[55] Hu Y (2016). Segregation between the parietal memory network and the default mode network: Effects of spatial smoothing and model order in ICA. Sci. Bull..

[56] Bruner E (2014). Midsagittal brain variation and MRI shape analysis of the precuneus in adult individuals. J. Anat..

[57] Baggio HC, Junqué C (2019). Functional MRI in Parkinson’s disease cognitive impairment. Int. Rev. Neurobiol..

[58] Lucas-Jiménez O (2016). Altered functional connectivity in the default mode network is associated with cognitive impairment and brain anatomical changes in Parkinson’s disease. Parkinson Relat. Disord..

[59] Kehagia AA, Barker RA, Robbins TW (2012). Cognitive impairment in Parkinson’s disease: The dual syndrome hypothesis. Neurodegener. Dis..

[60] Elena P, Demetris S, Christina M, Marios P (2021). Differences between exergaming rehabilitation and conventional physiotherapy on quality of life in Parkinson’s disease: A systematic review and meta-analysis. Front. Neurol..

[61] Shulman LM (2010). The clinically important difference on the unified Parkinson’s disease rating scale. Arch. Neurol..

